# Epigenetically Regulating Non-coding RNAs in Colorectal Cancer: Promises and Potentials

**DOI:** 10.34172/mejdd.2025.404

**Published:** 2025-01-31

**Authors:** Zahra Taheri, Majid Zaki-Dizaji

**Affiliations:** ^1^Department of Biology, Science and Research Branch, Islamic Azad University, Tehran, Iran; ^2^Human Genetics Research Center, Baqiyatallah University of Medical Sciences, Tehran, Iran

**Keywords:** Colorectal cancer, Non-coding RNA, Epigenetic, DNA methylation, Epitranscriptome, RNA modification

## Abstract

Colorectal cancer (CRC) is a common malignancy with high mortality. Despite advancements in understanding its molecular causes and improved drug therapies, patient survival rates remain low. The main reasons for the high mortality rate are cancer metastasis and the emergence of drug-resistant cancer cell populations. While genetic changes are recognized as the main driver of CRC occurrence and progression, recent studies suggest that epigenetic regulation is a crucial marker in cancer, influencing the interplay between genetics and the environment. Research has shown the significant regulatory roles of non-coding RNAs (ncRNAs) in CRC development. This review explores epigenetically regulated ncRNAs and their functions, aiming to understand key regulatory mechanisms that impact CRC development. Additionally, it discusses the potential use of these ncRNAs in CRC diagnosis, prognosis, and targeted treatments.

## Introduction

 Colorectal cancer (CRC), also known as colon or rectal cancer, is a common and aggressive form of cancer. It ranks as the fourth leading cause of cancer-related death globally, following lung, liver, and stomach cancer.^1,2^ Most patients either have metastases upon diagnosis or develop them later due to the natural progression of the disease.^3^ Despite significant progress in radiotherapy, chemotherapy, and surgical procedures for CRC, as well as improvements in screening programs and medical technologies, the overall survival rate of patients with CRC remains relatively low. Thus, a clinical imperative exists to enhance our understanding of the biological processes in CRC that lead to gene deregulation, tumor heterogeneity, and evasion of drug treatment effects. It is essential to identify new disease determinants and utilize them as biomarkers for early disease detection, predicting drug responses, and prognosis.^4^

 CRC is mostly a complex disease influenced by genetic and epigenetic risk factors and environmental factors that can impact its progression.^5^ Increasing evidence indicates that epigenetic changes play a role in shaping both normal physiological processes and the development of diseases, particularly in carcinogenesis.^6^ Epigenetic genome modifications are dynamic and reversible, including DNA, RNA, and chromatin modifications.^6-8^ We know that over 90% of the human genome is actively transcribed. Nevertheless, only 2% of these transcripts code for proteins, with the majority being non-coding RNAs (ncRNAs).^9^ These ncRNAs include microRNA (miRNA), small nuclear RNA (snRNA), small nucleolar RNA (snoRNA), piwi-interacting RNA (piRNA), long non-coding RNAs (lncRNAs), ribosomal RNA (rRNA), transfer RNA (tRNA), and circular RNA (circRNA).^10-13^ Also, two new classes of ncRNAs, known as promoter-associated RNAs (PARs) and enhancer RNAs (eRNAs), have recently been identified.^14^ The impact of ncRNAs in CRC is well-known,^15^ and these ncRNAs can be epigenetically regulated during CRC development, metastasis, and drug.^7^

 Because of the significance of epigenetic mutations (epimutations) in CRC and the increasing evidence of how epigenetically regulated ncRNAs contribute to CRC development, progression, and resistance, our objective is to outline recent discoveries, assess their molecular roles, and consider their potential as biomarkers for diagnosis, prognosis, and therapy.

## Epigenetically Regulated MicroRNA Markers in CRC

**Figure 1:**
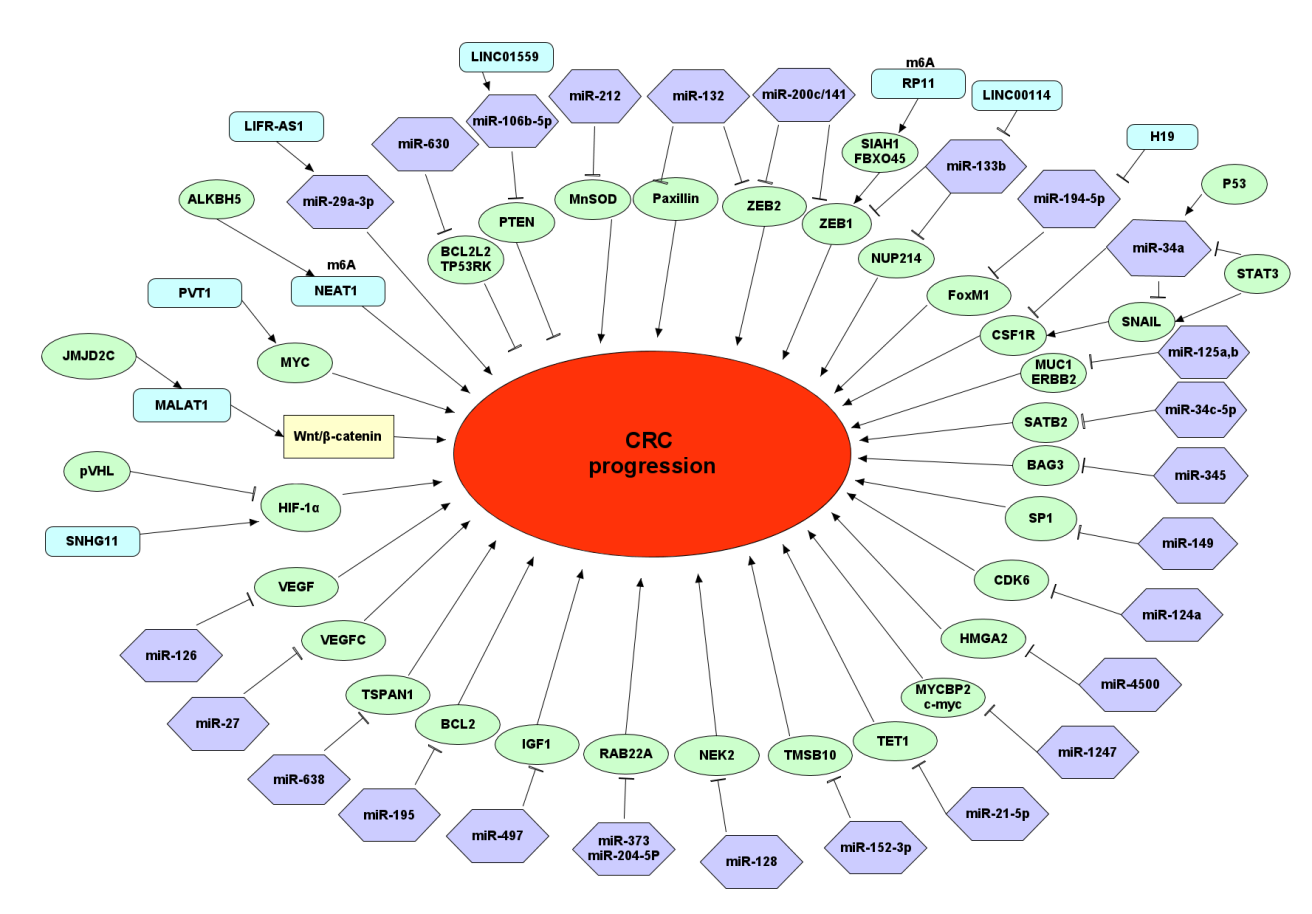
Impact of epigenetically regulated non-coding RNAs on colorectal cancer molecular pathways. The blue rectangles show lncRNAs, the green circles show genes, the purple hexagons are miRNAs, and the yellow rectangles are pathways. N6-methyladenosine (*m6A*)

 MicroRNAs are short, ncRNAs, typically 20-25 nucleotides in length, that are crucial in biological processes such as regulating gene expression and various cellular processes like cell proliferation, cycle cell regulation, apoptosis, and differentiation.^16-18^ MiRNAs are present in all tissues, most binding to specific target mRNAs through the 3′ UTR to downregulate gene expression or inhibit translation. However, reports suggest that miRNAs interact with various regions, such as promoters, the 5′ UTR, and the coding sequence.^16^ Additionally, microRNAs serve dual purposes as both oncogenes and tumor suppressors, playing a crucial role in the development of tumors.^19^ Dysregulation of miRNAs is linked to numerous human diseases.^20,21^ Epigenetic regulation, specifically DNA methylation, plays a crucial role in suppressing miRNAs. The abnormal methylation of miRNAs is a new type of biomarker that shows promise for diagnosing and prognostic markers in CRC. Recent studies have identified them as potential biomarkers for diagnosis and prognosis, as listed in Table 1 and Figure 1. Here, we discuss recently reported methylated miRNAs and their impact on CRC progression.

**Table 1:** Epigenetically regulated miRNAs in colorectal cancer

**miRNA Expression/ pattern in CRC**	**Target gene/ signaling pathway**	**Oncogene or Tumor-suppressor /biomarker**	**Sample type**	**Findings**	**Ref**
miR-124a↓	CDK6	TS / Prognostic	cell lines and 208 CRC	The hypermethylation of miR-124a results in the activation of the CDK6 oncogene and phosphorylation of Rb.	2007 ^22^
miR-342 ↓	-	TS	42 CRCs, 9 A, and 16 N	Methylated EVL/miR-342 was identified in a majority of CRCs, which suggests that it is an early event in CRC carcinogenesis.	2008 ^23^
miR-34b/c ↓	MET, CCNE2, SFRS2 and CDK4	TS	111 CRC and cell lines	The CpG island of miR-34b/c acts as a bidirectional promoter controlling the expression of different tumor suppressor genes like BTG4. This region is often methylated in CRC.	2008 ^24^
miR-345 ↓	BAG3	TS	CRC cells line	Low expression of mir-345 was associated with lymph node metastasis and worse histological type. Mir-345 acts as a growth inhibitor in CRC by targeting the BAG3 oncogene. This suggests its potential antineoplastic role in the development of CRC.	2011 ^25^
miR-373 ↓	RAB22A	TS	CRC cell lines and 40 CRC	miR-373 downregulated and RAB22A upregulated in CRC	2011 ^26^
miR-149 ↓	SP1	TS / Prognostic	86 CRC tissues and cell lines	Silencing miR-149 through methylation leads to the upregulation of Sp1, promoting CRC.	2013 ^27^
miR-34a ↓	c-Met, Snail, β-catenin, CD44^28^ in PC, Axl, TPD52, Lef1 and MTA2^29^	TS / Prognostic	94 CRC w/o liver metastasis	Hypermethylation of miR-34a causes elevated levels of c-Met, Snail, and β-catenin, which are linked to liver metastasis in CRC.	2013 ^30^
miR-497/195 ↓	miR-497: IGF1 ^31^ miR-195: BCL2 ^32^	TS	CRC cell lines and 50 polyps with PANS	Both miRNAs are hypermethylated and expressed at lower levels in CRC. The tumor-suppressor activity of miR-497 in CRC is achieved by reducing the expression of IGF1. miR-195 targets BCL2, and the decreased expression of miR-195 has been strongly associated with higher mortality rates in CRC patients.	2013 ^33^
miR-27↓	VEGFC	TS	CRC cell lines	miR-27b, found in CRC stem cells, functions as a crucial tumor suppressor and angiogenic factor by targeting VEGFC.	2013 ^34^
miR-212 ↓	MnSOD	TS / Prognostic	180 CRC with PANS and cell lines	miR-212 inhibits metastasis and EMT in CRC by targeting MnSOD. The low level of miR-212 is linked to aggressive tumor behavior and a negative disease progression.	2013 ^35^
miR-126 ↓	VEGF	TS / Therapeutic target	12 CRC with PANS 62 CRC and cell lines	miR-126 directly silences VEGF expression, leading to the inhibition of cell invasion and tumor angiogenesis in CRC.	2013 ^36^
miR-638 ↓	TSPAN1, SOX2 ^37,38^ Sp2^39^ in GC	TS / Prognostic	cell lines and 156 CRC with PANS	Downregulation of miR-638 in CRC was associated with poor prognoses. miR-638 inhibited CRC cell growth, invasion, and cell cycle progression by targeting TSPAN1.	2014 ^40^
miR-204-5p↓	RAB22A	TS / Prognostic	CRC Cell lines and 272 CRC with PANS	Downregulation of miR-204-5p in CRC was associated with poor prognoses. miR-204-5p plays a role in inhibiting EMT.	2014 ^41^
miR-128 ↓	NEK2	TS / Prognostic	180 CRC and cell lines	MiR-128 inhibited NEK2 expression and cancer cell proliferation via cell cycle arrest. High miR-128 expression is associated with a low recurrence rate.	2014 ^42^
miR-132 ↓	Paxillin, ZEB2 ^43^	TS / Prognostic	36 CRC with PANS and cell lines	Downregulation of miR-132 may occur as a result of hypermethylation and implies a poor prognosis in CRC. miR-132 suppresses cell invasion and EMT in CRC by directly targeting ZEB2.	2015 ^44^
miR-125a,b ↓	MUC1, ERBB2, and ERBB3 in BC ^45^	TS / Prognostic	68 CRC with PANS	Hypermethylation of miR-125 was found to have a negative impact on the clinical outcome of patients.	2015 ^46^
miR-133b↓	-	TS	CRC cell lines and CRC tissues	Methylation of miR-133b disrupts apoptosis, cell cycle progression, and invasion in CRC cells.	2015 ^47^
miR-4500 ↓	HMGA2	TS / Prognostic	75 CRC with PANS	Downregulated miR-4500 indicated an advanced tumor stage and poor survival. In vitro, miR-4500 suppressed CRC cell proliferation, migration, and invasion, while in vivo, it inhibited tumor growth by targeting HMGA2.	2016 ^48^
miR-23b ↓	PLAU, c-met ^49^ in HCC SMADs/TGF- β/BMP^50^ in liver stem cells	TS / Diagnostic and Prognostic	96 CRC plasma	Decreased levels of plasma miR-23b were strongly linked to clinical stage, tumor depth, distant metastasis, and tumor recurrence and ultimately shorter recurrence-free survival times and lower overall survival rates.	2016 ^51^
miR-1247 ↓	MYCBP2	TS / Therapeutic target	35 CRC and cell line	miR-1247 suppresses tumor growth by targeting the oncogene MYCBP2 and its downstream c-myc in methylator CRC, effectively inhibiting tumor progression.	2018 ^52^
miR-21-5P ↑	TET1 PTEN, PDCD4, SPRY2^53^	Onco / Diagnostic and Prognostic	164 CRC and cell lines	TET1 acts as a suppressor of tumor growth and inhibits EMT. miR-21-5P directly targets TET1, leading to the promotion of EMT, migration, and invasion in CRC.	2018 ^54^
miR-34c-5p ↓	SATB2	TS / Prognostic	85 CRC with PANS and cell lines	miR-34c-5p targets SATB2, reducing metastasis and inhibiting EMT in CRC.	2018 ^55^
miR-137 ↓ miR-342↓	-	TS / Diagnostic and Prognostic	51 polyps, 8 CRC, and 14 N	The methylation frequency of miR-342 was higher than miR-137	2019 ^56^
miR-200c/141 ↓	ZEB1, ZEB2 ^57^	TS / Prognostic	34 CRC, 60 polyps with PANS, 20 N	DNA methylation of the miR-200c/141 cluster correlated with tumor stage and poor prognosis. The miR-200c-141 cluster is crucial in inhibiting EMT by targeting ZEB1 and ZEB2.	2019 ^58,59^
miR-152-3p↓	TMSB10	TS / Prognostic	88 CRC	DNMT1 and TMSB10 upregulated and miR-152-3p downregulated in CRC. DNMT1 maintained methylation of miR-152-3p	2020 ^60^
miR-34a ↓	CSF1R, PDGFR^61,62^	TS	CRC cell lines	P53 suppresses CRC invasion, EMT, and metastasis partly through downregulation of CSF1R by inducing miR-34a. CSF1R is directly and indirectly induced by SNAIL.	2020 ^63^

## MiRNA-124

 MiR-124 is an 85-base miRNA found on the minus strand of chromosome 8p23.1. It is predominantly expressed in the CNS and plays a crucial role in synaptic transmission, neuronal differentiation, stem cell regulation, and gastrulation.^22,64,65^ Researchers have found that miR-124 plays a tumor-suppressing role in different types of cancers, including colorectal and lung cancer. This is due to its specific methylation in tumors, low expression, hypomethylation in normal tissues, and high expression.^22,66^ Additionally, miR-124 is the initial microRNA in CRC that has been proven to be silenced through an epigenetic process.^22,67^ When the epigenetic mechanism leads to hypermethylation of CpG island in promoter miR-124a, it leads to the activation of an oncogene called CDK6 and the phosphorylation and suppression of a tumor suppressor gene known as Rb. CDK6 is essential for cell cycle progression and differentiation. Its suppression through miR-124 could serve as a valuable biomarker for cancer prognosis and the development of anticancer treatments.^22^ Studies have shown that miR-124 methylation in bowel lavage fluid (BLF) is altered in patients with CRC, suggesting methylated miR-124-3 may be a potential non-invasive diagnostic biomarker for CRC.^68^ Zhou and co-workers showed that reducing miR-124 levels could lead to increased cell growth, movement, invasion, and spread in CRC by inhibiting ROCK1 expression.^66^ This evidence shows that miR-124 serves as a prognostic biomarker in patients with CRC.

## MiR-126

 MiR-126 is recognized as a key regulator of angiogenesis. New studies have uncovered conflicting roles of miR-126 in cancer development. Research has demonstrated that miR-126 functions as a tumor suppressor by reducing tumor cell growth and spreading by targeting oncogenes like ADAM9, SLC7A5, and Crk.^36^ In contrast, recent studies suggest that miR-126 can play an oncogenic role by encouraging gastric carcinogenesis by suppressing SOX2 expression.^69^ The luciferase reporter assay showed that miR-126 directly binds to the 3’UTR of VEGF mRNA, leading to the inhibition of cell migration, invasion, and tumor neovascularization caused by CRC cells.^70^ Recent studies have indicated a reduction in miR-126 expression in CRC.^71^ Bioinformatic prediction tools have shown a conserved binding site for miR-126 in the 3’UTR of VEGF mRNA. The findings indicate that the silencing of miR-126 through promoter methylation is a significant factor in the disruption of VEGF expression in CRC. Therefore, miR-126 could potentially be a prognostic biomarker and targeted for CRC treatment.

## MiR-1247

 MiR-1247 is a novel miRNA that acts as a tumor suppressor and is found on the minus strand of 14q32.31. New research has found that MYC binding protein 2 (MYCBP2) is a gene targeted by miR-1247 in colon cancer. MYCBP2, is a protein that binds directly to the proto-oncogene c-myc and is involved in differentiation, cellular proliferation, and apoptosis. The specific molecular mechanisms triggered by MYCBP2 remain largely unidentified, but a significant finding is a substantial reduction in c-myc expression observed in cells with miR-1247 overexpression^52,72,73^ Liang and colleagues conducted a study using tumor samples from patients with hypermethylated and non-methylated colon cancer, as well as cell lines. They discovered a correlation between MYCBP2 protein levels, miR-1247 levels, and patient survival.^33^ Overall, DNA hypermethylation silences miR-1247, allowing MYCBP2 and c-myc protein to increase and promote tumor growth in CRC. So, the connection between MYCBP2, c-myc, and miR-1247 could be key in fighting tumors. Targeting this axis with demethylation agents may offer a potential treatment option.

## MiR-212

 MiR-212 is an intronic miRNA located on the distal end of chromosome 17 at p13.3. It is highly conserved in vertebrates and is generated from a stable intron of a non-protein coding gene. Several studies have shown that this specific miRNA acts as a tumor suppressor and is downregulated in various types of cancers, including gastric, non-small cell, and lung cancers.^35^ Furthermore, a separate study indicated that miR-212 could serve as a prognostic biomarker in acute myeloid leukemia.^74^ The expression of miR-212 is reduced in human CRC tissues due to genetic and epigenetic factors such as promoter hypermethylation.

 While MECP2, PTCH1, and PED have been previously recognized as targets of miR-212, Meng and others conducted bioinformatic analysis and experiments that revealed MnSOD as another direct target of miR-212 in CRC. They found that the 3′UTR of MnSOD serves as the functional target site for miR-212.^35^ MnSOD is an antioxidant enzyme found in the mitochondrial matrix. It may have important implications in the development of cancer.^75^ The process of EMT plays a critical role in the spread and growth of CRC cells.^76^ MnSOD is a key player in this process. They used western blot analysis to measure the expression levels of MnSOD and miR-212. They discovered that a decrease in MnSOD and overexpression of miR-212 resulted in an increase in epithelial markers and a reduction of mesenchymal markers. In summary, the findings indicate that miR-212 suppresses EMT in CRC cells by repressing MnSOD activity.^35^

 The reduction of miR-212 could potentially serve as a prognostic marker for patients with CRC, as it might prevent tumor progression by targeting MnSOD messenger RNA. Both miR-212 and MnSOD could also be considered therapeutic targets for cancer treatment.

## MiR-128

 Takahashi and co-workers discovered that miR-128 is often reduced in advanced CRC due to increased promoter hypermethylation. Furthermore, the decrease in miR-128 levels was strongly linked to higher recurrence rates in CRC. miR-128 directly targets NEK2, causing G2-phase cell cycle arrest and suppressing cancer cell growth. Additionally, it is epigenetically silenced in CRC cells. High levels of NEK2 in CRC tissues were linked to a negative prognosis. The miR-128/NEK2 pathway could be a promising therapeutic target for individuals with CRC.^42^

## MiR-373

 Tanaka and colleagues analyzed miRNA expression in CRC cell lines pre- and post-5-aza-2’-deoxycytidine (DAC) treatment. They identified 10 miRNAs with more than a 2-fold increase after DAC treatment in each cell line. Specifically, they focused on miR-373 and discovered that its overexpression inhibited cell proliferation. Furthermore, they found that miRNA expression was repressed due to abnormal methylation in colon cancer cell lines. miR-373 serves varying roles in different types of malignant tumors, acting as a tumor suppressor in CRC. Computational predictions have identified RAB22A as a potential target gene for miR-373. In contrast, RAB22A functions as an oncogene with increased expression levels in CRC and malignant melanoma.^77^ In clinical samples showing abnormal methylation of the miR-373 promoter region, the expression of miRNA was decreased, while the levels of the RAB22A target gene were elevated. This study revealed that silencing miR-373 plays a critical role in the progression of CRC.^26^

## Other miRNAs Genes

###  miR-133b and miR-1

 MiR-133b acts as a tumor suppressor gene in CRC and is frequently silenced by CpG methylation in the promoter region.^78^ Surprisingly, miR-133b actually inhibits the HOXA9/ZEB1 pathway, leading to an increase in tumor metastases and worse outcomes in CRC.^79^ Furthermore, DNA hypermethylation of miR-1 was initially identified in hepatocellular carcinoma (HCC) and later found in CRC. On the other hand, miR-1 interacts with miR-133a in CRC, and silencing both microRNAs has a negative effect on TAGLN2 expression. The interaction between miR-1 and miR-133a, leading to the upregulation of TAGLN2, plays a crucial role in CRC.^80^

## Epigenetically Regulated Long Non-coding RNAs in CRC

 LncRNAs are RNA molecules have over 200 nucleotides and cannot encode proteins^81,82^ lncRNAs play a role in various cellular processes, such as gene regulation and chromatin dynamics. They are also involved in important functions like cell proliferation, differentiation, and apoptosis.^83^ Some reports have shown that lncRNAs play a crucial role in the development and advancement of various types of tumors.^84^ We explored the epigenetically directed aberrant lncRNAs expression and their possible roles in the development and advancement of CRC (Table 2). These findings suggest that lncRNAs could serve as valuable markers for diagnosis and prognosis.

**Table 2:** Epigenetically regulated lncRNAs in colorectal cancer

**lncRNA/ Expression pattern in CRC**	**Epigenetic regulation**	**Target gene/signaling pathway**	**Oncogene or tumor-suppressor/ biomarker**	**Sample type**	**Findings**	**Ref**
CAHM↓	DNA HypeM	-	TS/ Diagnostic	Tissue: 26 N, 21 A, 87 AC Plamsa: 74 N, 73 A, 73 CRC	Methylated CAHM has been found in patients' plasma and tissue, suggesting a possible role in non-invasive CRC detection assays.	2014 ^85^
TUG1↑	HDAC1	E-cadherin**↑** and N-cadherin, vimentin, and Fibronectin**↓**	Onco/ Prognostic	120 CRC with PANS, cell lines	The high levels of TUG1 in CRC show poor prognosis, leading to lower survival rates and an increased risk of cancer metastasis. TUG1 regulates the invasive and metastatic capabilities of CRC cells, in part through the modulation of EMT.	2016 ^86^
LINC00114 ↑	DNA HypoM	miR-133b	Onco/ Diagnostic	CRC cell lines	LINC00114 regulates the expression of the NUP214 protein by sponging miR-133b. LINC00114 inhibits miR-133b expression through the methylation of its promoter region by the EZH2/DNMT1 complex.	2019 ^87^
H19 ↑	DNA HypoM	miR-194-5p	Onco/ Diagnostic	214 CRC with PANS, cell line	H19 inhibits miR194-5p, affecting the expression of FoxM1 and regulating the metastasis and EMT of CRC cells.	2019 ^88^
LINC00460 ↑	DNA HypoM	-	Onco/ Prognostic	407 CRC tumors and 21 ANS	LINC00460 hypomethylation and expression promote CRC metastasis and are associated with poor survival rates in CRC patients.	2019 ^89^
RP11 ↑	m6A methylation	SIAH1 & FBXO45	Onco/ Prognostic	CRC cell lines & tumor tissue	The expression of RP11 was significantly higher in CRC. RP11 played a crucial role in the metastasis of CRC cells by regulating Siah1-Fbxo45/Zeb1.	2019 ^90^
MALAT1 ↑	DNA HypoM	β-catenin signaling pathway, AKAP-9	Onco/ Prognostic	78 CRC tissue and CRC cell lines	JMJD2C enhances the metastatic abilities of CRC cells by regulating the histone methylation level of MALAT1 promoter, thereby upregulating the expression of MALAT1 and enhancing the activity of β-catenin signaling pathway.	2019 ^91^
NEAT1 ↑	m6A demethylation	-	Onco/ Prognostic	70 CRC tissues and PANS	NEAT1 levels significantly increased in CRC tissues, correlated with poor prognosis. ALKBH5 facilitated the upregulation of NEAT1 expression through demethylation.	2020 ^92^
SNHG11 ↑	DNA HypoM	HIF-1α/AK4, ENO1, HK2, and Twist1	Onco/ Prognostic	164 CRC with PANS	SNHG11 inhibits the binding of pVHL to HIF-1α by occupying the recognition sites. This action promotes migration and invasion in CRC cells by activating downstream targets of HIF-1α.	2020 ^93^
PVT1 ↑	DNA HypoM	MYC, TGFβ/SMAD and Wnt/β-Catenin pathways	Onco/ Prognostic	426 CRC patients, CRC cell line	PVT1 enhances the oncogenic potential of MYC through epigenetic regulation. PVT1 locus could impact the expression of TGFβ/SMAD and Wnt/β-catenin pathways genes.	2020 ^94^
LINC00152 ↑	DNA HypoM	Cyclin D1, PI3K/Akt, Ras, WNT, TP53, Notch and ErbB	Onco/ Prognostic	43 N, 55 A, 43 CRC	LINC00152 significantly upregulated in CRC by promoter hypomethylation. LINC00152 contributes to CRC progression through PI3K/Akt, Ras, WNT, TP53, Notch, and ErbB.	2020 ^95^
LIFR-AS1 ↓	DNA HyperM	miR-29a-3p	TS/ Diagnostic	92 CRC tissues and 43 normal tissues	DNA hypermethylation causes a decrease in LIFR-AS1, leading to the advancement of CRC. Its downregulation is associated with poor prognosis.	2022 ^96^
LINC01559 ↓	DNA HypoM	miR-106b-5p	TS	Fresh CRC tissues and PANS	LINC01559 was downregulated in CRC and associated with poor prognosis. LINC01559 upregulates PTEN through sponging miR-106b-5p. LINC01559/miR-106b-5p/PTEN axis is a negative regulation of CRC.	2022 ^84^

AP-2α; activator protein 2α, MDR; multidrug resistance, PANS: paired adjacent normal specimens, EMT: epithelial-to-mesenchymal transition, PANS: paired adjacent normal specimens, A: adenomas, N: normal, AC: adenocarcinoma, TS: tumor suppressor, onco: oncogene.

## Small Nucleolar RNA Host Gene 11 (SNHG11) lncRNA

 SNHG11 is an intergenic lncRNA found on the plus strand of chromosomal 20q11.23. It has a length of 4598 nt and is composed of five exons^97^ SNHG11 is a key player in promoting the invasion and metastasis of CRC cells while inhibiting apoptosis. Xu and colleagues^93^ discovered that SNHG11 lncRNAs were likely regulated by DNA methylation in The Cancer Genome Atlas (TCGA)-COAD, highlighting its significance in CRC. This suggests that DNA methylation could influence SNHG11 expression, as it is upregulated due to promoter hypomethylation in CRC. SNHG11 knockdown was found to inhibit the migration and invasion of CRC cells under hypoxic conditions. HIF-1α stabilization is crucial for cells adapting to changes in oxygen levels. This process is closely monitored by factors like PHD, pVHL, and ncRNAs like miR-200b, miR-200c, and miR-429. SNHG11 binds to specific sites on HIF-1α, preventing its degradation by blocking the interaction with pVHL. Increased levels of HIF-1α in CRC promote metastasis by controlling various target genes. Additionally, SNHG11 boosts the expression of HIF-1α target genes like AK4, ENO1, HK2, and Twist1.^93,98^

 In summary, this finding shows that the lncRNA SNHG11 boosts the stability and activity of HIF-1α in CRC cells, leading to increased invasion and metastasis. SNHG11 may be used as a prognostic marker and treatment target for patients with CRC.

## LINC00460

 Zhang and co-workers have noted that abnormal lncRNA expression significantly influences various biological processes in CRC, including tumor growth, spread, and proliferation. By analyzing the TCGA database, they pinpointed LINC00460 as the most commonly activated lncRNA in patients with CRC compared with healthy tissues.^89^ LINC00460 is 9936 nucleotides long and located on the plus strand of chromosome 13q33.2.^97^ It is suggested that this gene may have an oncogenic role in cancer and potentially exert a carcinogenic effect. Previous studies have demonstrated that the overexpression of LINC00460 is linked to increased cell proliferation and invasion in various types of cancer, such as gastric cancer, lung cancer, ovarian cancer, and esophageal cancer. Despite its unclear role in colon cancer, Zhang’s results suggest that inhibiting the LINC00460 gene can slow down the proliferation of CRC cells, pointing to its potential carcinogenic effect on tumor growth in CRC. In conclusion, research studies in vitro and in living organisms have shown that the LINC00460 lncRNA is upregulated in CRC, triggered by DNA methylation. This gene is linked to tumor spread, promoting invasion and migration of CRC cells, which could impact patient prognosis.^89^

## LIFR-AS1

 LIFR AS1, located on chromosome 5p13.1, is a new long ncRNA that acts as a tumor suppressor in CRC. It is transcribed in an antisense manner from the LIFR gene. Abnormal expression of LIFR-AS1 has been observed in various human tumors.^96^

 Zhang’s research indicates that abnormal DNA methylation leads to the decreased expression of LIFR-AS1, which in turn promotes the advancement of colon cancer. They found increased methylation of a CpG island in the promoter region of LIFR-AS1, accelerating cancer progression. The methylation level in LIFR-AS1 demonstrated high sensitivity and specificity in diagnosing CRC. Functional tests conducted by Song revealed that LIFR-AS1 can competitively bind to hsa-miR-29b-3p, inhibiting the proliferation, colony formation, and invasion of colon cancer cells.^96^ Furthermore, Liu et al. found in a separate study that LIFR-AS1 functions as a sponge for miR-29a in CRC. Knocking down LIFR-AS1 diminished the impact of photodynamic therapy (PDT) on the proliferation and apoptosis of CRC cells, suggesting that LIFR-AS1 may act as a tumor suppressor by interacting with miR-29a.^99^ Furthermore, high levels of SNRPF, which is indirectly associated with LIFR-AS1, were observed in CRC cells. Increased SNRPN expression was found to be indicative of a poor prognosis.^96^ These results showed that the methylation level of LIFR-AS1 is highly sensitive and specific for diagnosing CRC and is also linked to the prognosis of the disease.

## NcRNA and Drug Resistance

 Although treatments like chemotherapy, targeted therapy, and immunotherapy have improved patient survival in CRC, the development of primary and secondary drug resistance poses a significant clinical challenge. The heterogeneity of CRC and the issue of drug resistance continue to hamper effective cancer treatment. Epigenetic modifications, which can be present in circulating tumor cells, play a key role in these challenges.^100^ Therefore, targeting epigenetic regulators is now seen as a promising strategy to overcome drug resistance.^101^

**Figure 2:**
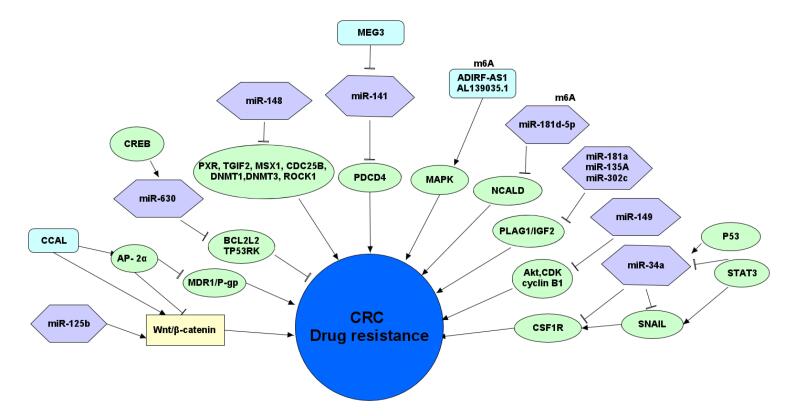
Role of epigenetically regulated non-coding RNAs in drug resistance in colorectal cancer

 The role of ncRNAs in drug resistance is increasingly recognized.^102^ METTL3-dependent m6A methylation of miR-181d-5p ^103^ by directly targeting neurocalcin δ (NCALD) inhibits the 5-FU sensitivity of CRC cells. Also, this modification in lncRNA ADIRF-AS1 and AL139035.1 regulates 5-FU drug resistance formation through MAPK signaling.^104^ Despite m6A methylation, DNA hypermethylation or hypomethylation in different miRNA and lncRNA have been shown to contribute to drug resistance (Table 3, Figure 2).

**Table 3:** The role of epigenetically regulated non-coding RNA in drug resistance

**Expression**	**Epigenetic**	**Target gene/ signaling pathway**	**Oncogene or Tumor-suppressor /biomarker**	**Sample type**	**Biological function**	**Ref**
miR-148a↓	HyperM.	PXR, TGIF2, MSX1, CDC25B, DNMT1, DNMT3 and ROCK1	TS / 5-FU and oxaliplatin	273 CRC patients (76 stage II, 125 stage III, 72 stage IV)	miR-148a expression was down-regulated in advanced CRC tissues, associated with poor prognosis and poor response to 5-fluorouracil and oxaliplatin-based chemotherapy.	2012 ^105^
miR-630 ↑	DNA HypoM	BCL2L2 and TP53RK	TS/radiosensitivity	CRC cell lines	miR-630 expression positively correlated with radiosensitivity. Methylation and CREB modulated miR-630 expression. CREB–miR-630–BCL2L2 and TP53RK pathway regulate radiosensitivity.	2015 ^106^
CCAL↑	HypoM	AP-2α and MDR1/P-gp	Onco/ MDR	252 CRC with PANS	CCAL enhances CRC progression and multidrug resistance by activating the Wnt/β-catenin signaling through targeting AP-2α and, in turn, MDR1/P-gp, respectively. Patients with high CCAL expression show shorter overall survival and worse response to adjuvant chemotherapy.	2016 ^107^
MIR100HG↑ miR-100 ↑ miR-125b↑	HypoM	Wnt/β-catenin negative regulators	Onco / cetuximab-resistant	CRC cell lines	MIR100HG-derived miR-100 and miR-125b mediate cetuximab resistance via Wnt/β-catenin pathway. GATA6 represses MIR100HG, but this repression is relieved by miR-125b targeting of GATA6.	2017 ^108^
miR-181a↓ miR-135a↓ miR-302c↓	HyperM	PLAG1/IGF2 signaling	TS / 5-FU	67 CRC, and cell lines	miR-181a/135a/302c function as tumor suppressors via repressing PLAG1/IGF2 signaling. Their expression promoted the sensitivity of CRC cells to 5-FU treatment.	2018 ^109^
MEG3↓	HyperM^110^	miR-141/ PDCD4	TS/oxaliplatin	48 CRC with PANS, cell lines	Low MEG3 expression was correlated with poor prognosis. MEG3 was down-regulated in oxaliplatin-resistant CRC tissues and cell lines. MEG3 elevated PDCD4 expression through targeting miR-141	2018 ^111^
miR-34a↓	HyperM	CSF1R, SNAIL	TS / 5-FU	CRC cell lines	CpG-methylation of miR-34a results in elevated expression of CSF1R and 5-FU resistance. High CSF1R expression is associated with poor prognosis and metastasis.	2020 ^63^
miR-149 ↓	HyperM	Akt, cyclin B1, CDK	TS / MDR	CRC cell lines	Hypomethylation of the miR-149 CpG island and upregulation triggers cell cycle arrest by reducing the expression of AKT, Cyclin B1, and CDK1. Thus, leads to improved sensitivity to chemotherapy in CRC.	2021 ^112^
miR-181d-5p	m6A methylation	NCALD	TS / 5-FU	141 CRC tissues and FFPE	METTL3-dependent m6A methylation was upregulated in CRC to promote the processing of miR-181d-5p. This led to increased miR-181d-5p expression, which inhibited the 5-FU sensitivity of CRC cells by targeting NCALD.	2022 ^103^
ADIRF-AS1 AL139035.1	m6A methylation	MAPK signaling	onco / 5-FU	CRC cell lines	ADIRF-AS1 and AL139035.1 promote CRC progression and may regulate drug resistance through MAPK signaling (FOS, DUSP1, MEF2C).	2024 ^104^

MDR: Multidrug resistance; EMT: epithelial-to-mesenchymal transition; PANS: paired adjacent normal specimens; A: adenomas; N: normal.

## LncRNA Colorectal Cancer-Associated lncRNA (CCAL)

 CCAL an oncogenic lncRNA, actively promotes the development and advancement of CRC. Several studies have shown that CCAL significantly contributes to the progression of various tumors, particularly CRC. Ma and others investigated the oncogenic properties and impact of CCAL on CRC. They discovered that epigenetic regulatory factors, like DNA methylation, control the expression of CCAL. Additionally, through methylation-specific PCR analysis, they observed a lower level of methylation in the CpG island region of CCAL in CRC tissue samples compared to normal tissue samples. CCAL is one of lncRNAs that plays a crucial role in regulating molecular pathways through its interactions with proteins ^107,113^ According to Ma and colleagues, high levels of CCAL can trigger cell proliferation, invasion, cell-cycle progression, migration, and invasion in CRC by inhibiting the AP-2α protein. Additionally, CCAL activates the Wnt/β-catenin pathway by suppressing AP-2α. Conversely, reducing CCAL levels results in increased AP-2α expression, decreased β-catenin expression, and elevated levels of c-myc, cyclin D1, and E-cadherin. Furthermore, CCAL plays a role in regulating MDR1/P-gp expression by activating the Wnt signaling pathway. MDR is a significant challenge in successful chemotherapy for patients with CRC. In summary, lncRNA-CCAL controls CRC progression and MDR by activating the Wnt/β-catenin signaling pathway, suppressing AP-2α, and increasing MDR1/P-gp expression.^107^ This finding indicates that lncRNA CCAL could serve as a valuable new prognostic biomarker for patients with CRC and advanced disease or metastasis.

## MiRNA-125

 The miR-125 family consists of two distinct members, miR-125a and miR-125b, which are located on separate chromosomes. The miR-125a molecule consists of 86 bases and is found on chromosome 19q13.41 on the plus strand. In contrast, miR-125b is an 88-base molecule located on chromosome 11q24.1 on the minus strand.^46^ Recent studies show that the miR-125 family is dysregulated in various types of human cancer, including gliomas, prostate cancer, breast cancer, and gastric cancer. Depending on the type of cell, both miR-125a and miR-125b can either promote cancer growth or suppress it. For example, in prostate cancer, miR-125b acts as an oncogene and promotes tumor growth by inhibiting the intrinsic apoptosis pathway by targeting PUMA, P53, and BAK.^114^ In contrast, miR-125b acts as a tumor suppressor in breast cancer by suppressing the oncoproteins MUC1, ERBB2, and ERBB3, thereby inhibiting tumor growth.^45^ However, miR-125a significantly reduced the growth, movement, and infiltration of cancer cells, including gastric and breast cancer^45,115^ While the role of miR-125 in CRC is not yet fully understood, a recent study revealed that both miR-125a and b are frequently reduced in CRC tissues by hypermethylation. This suggests that the miR-125 family may possess tumor-suppressing properties in CRC^46^ (Table 1). However, hypomethylation of lncRNA MIR100HG region and, consequently upregulation of miR-125b associated with cetuximab resistance via Wnt/β-catenin pathway^108^ (Table 3), and depression of miR-125b-2-3p associated with cell sensitivity to first-line chemotherapy (fluorouracil, oxaliplatin, CPT-11).^116^

## MiR-34

 The miR-34 family consists of three members, namely miR-34a, miR-34b, and miR-34c, which are encoded by genes located on chromosomes 1 and 11. These miR-34 family members exhibit tumor suppressor properties by suppressing the expression of their target mRNAs.^117^ The regulation of miR-34 expression involves various mechanisms that contribute to its dysregulation in cancer. Studies have shown reduced expression of miR-34b/c,^24^ miR-34c-5p,^55^ and miR-34a^30,63^ in CRC through epigenetic mechanisms. The tumor suppressor protein p53 directly interacts with the miR-34 gene promoter, leading to the activation of its transcription. Specifically, the activation of miR-34a enhances the functions of p53, including cell cycle arrest, DNA repair, and apoptosis.^118^ Furthermore, TP53 gene polymorphisms have been linked to the methylation and expression levels of miR-34a/b/c in CRC tissues.^119^

 The miR-34 gene is targeted by activated STAT3, repressing miR-34 transcription and promoting EMT in CRC cells and tumors.^117^ Additionally, in CRC, the control of tyrosine kinase colony-stimulating factor 1 receptor (CSF1R) by p53-inducible miR-34a is disrupted due to a feedback loop involving STAT3. Shi and colleagues^63^ showed that miR-34a directly impacts CSF1R and plays a crucial role in the collaborative action of p53 and miR-34a in limiting CRC progression. P53 reduces CSF1R expression by upregulating miR-34a, while SNAIL increases CSF1R expression by downregulating miR-34a both directly and indirectly. CSF1R, when activated through a STAT3-mediated pathway, promotes EMT, migration, colonization, and metastasis in CRC cells. Methylation of CpG sites on miR-34a leads to increased expression of CSF1R, contributing to resistance to 5-FU in CRC cells (Table 3).

## lncRNAs Act as Competing Endogenous RNA (ceRNAs)

 In 2011, Pier Paolo Pandolfi’s group introduced the concept of a new RNA interaction mechanism known as ceRNA. This theory proposes that various types of RNAs, including coding RNAs and ncRNAs (like lncRNAs, circRNAs, and pseudogenes), communicate with each other through miRNA complementary sequences called MREs. This interplay creates a vast regulatory network within the transcriptome. Many lncRNAs play a key role in regulating gene expression by interacting with microRNAs through a process known as ceRNA mechanism.^120^ Previous research demonstrated that LIFR-AS1 functions as a ceRNA in various types of cancer. Specifically, LIFR-AS1 has been found to sponge miR-29a-3p and miR-4698 in gastric cancer, miR-150-5p in pancreatic cancer, miR-942-5p in lung cancer, miR-197-3p in breast cancer, miR-4262 in glioma and miR-31-5p in thyroid carcinoma.^96^

 In a study by Lin et al, LIFR-AS1 was identified as a ceRNA for miR-29a, which inhibits its expression and increases TNFAIP3 expression. This process helps regulate resistance to PDT in CRC. The researchers observed a negative regulatory relationship between LIFR-AS1 and miR-29a in PDT-treated HCT116 cells through direct binding. Knocking down LIFR-AS1 reduced the impact of PDT on CRC cell proliferation and apoptosis, suggesting LIFR-AS1 may function as a tumor suppressor by interacting with miR-29a.^54^ In addition, Song and colleagues conducted a study on the ceRNA function of LIFR-AS1 in CRC. They discovered that LIFR-AS1 can interact with hsa-miR-29b-3p using a luciferase reporter gene in colon cancer cells.^96^

 H19 is an oncofetal ncRNA that is hypomethylated and upregulated in CRC, promoting its development by generating miRNA or serving as ceRNA.^121^ H19 and miR-194-5p alter the EMT, invasion, and migration of CRC cells by targeting downstream FoxM1. FoxM1, influenced by H19 and miR-194, serves as an oncogene in CRC. H19 can regulate EMT-related genes by sponging miRNAs. In addition, FoxM1 can counteract the effects of miR-194-5p on suppressing invasion, migration, and EMT in CRA cells. Li and co-workers demonstrated the LncRNA H19/miR-194/FoxM1 axes could be a valuable target for diagnosing and treating CRC.^88^

 The lncRNA LINC00114 is associated with cancer lncRNA and is upregulated in CRC. Through DNA methylation, LINC00114 negatively regulates the expression of miR-133b, indicating its role as an oncogene in CRC development. Research has shown that miR-133b is crucial in advancing CRC as it inhibits cell growth and spread. NUP214 plays a crucial role in mitosis and cancer development, and it has been identified as a direct target of miR-133b. A study by Lv and others showed that LINC00114 can regulate the expression of the NUP214 protein by acting as a sponge for miR-133b. ^87^

## snoRNAs

 snoRNAs are a crucial class of ncRNAs that may undergo changes in human cancer. These RNAs are located in the nucleolus and play a significant role in various cellular functions, including RNA modification, pre-RNA processing, and the regulation of alternative splicing. Studies have suggested that snoRNAs could contribute to the development and progression of cancer. Ferreira and co-workers conducted research using Bisulfite genomic sequencing on multiple clones from normal colon mucosa and the CRC cell line hcT-116, revealing that certain snoRNAs were hypomethylated while others were hypermethylated.

 In cancer cells, snoRNAs SNORD123, U70c, and AcA59B, as well as the 5’-cpG islands associated with their host genes, were hypermethylated, which was not observed in the corresponding normal tissue. Recent research has shown that snoRNAs are frequently hypermethylated in different tumors, specifically in leukemias and CRC. This highlights the need for a more in-depth investigation of this specific group of ncRNAs that are affected by epigenetic changes in human cancer.^122^

## RNA Epitranscriptome

 Various chemical modifications occur on RNA bases and ribose molecules, playing a crucial role in the post-transcriptional regulation of gene expression. To date, various types of RNA modifications have been identified on both coding and predominantly ncRNA molecules. Similar to modifications found on DNA and histone proteins, RNA modifications can be added, removed, and recognized by specific enzymes. These modifications typically impact RNA processes such as splicing, stability, localization, translation, and interactions between RNA molecules and RNA-binding proteins, thereby influencing cellular activities.^123^

 Recent studies have highlighted the emerging role of RNA modifications in various cancers, including CRC^124,125^ Substantial evidence demonstrates the impact of m6A modification on the progression and development of drug resistance in CRC (Tables 2 and 3). One example of this is the upregulated expression of RP11 in CRC, which has been associated with m6A modification, leading to its localization to chromatin. The upregulation of RP11 stimulates the expression of Zeb1 by downregulating Siah1 and Fbxo45 mRNA expression as RP11 binds to hnRNPA2B1. This mechanism ultimately results in the degradation of Siah1 and Fbxo45, thereby preventing the degradation of the mesenchymal transition-related gene Zeb1. Zeb1, functioning as an epithelial-mesenchymal transition transcription factor (EMT-TF), plays a critical role in promoting EMT progression by specifically targeting E-Cadherin expression.^90^

 Additionally, elevated levels of NEAT1 have been observed in CRC tissues and are associated with a poor prognosis. The upregulation of NEAT1 expression is mediated by ALKBH5 through m6A demethylation.^92^ NEAT1 plays a role in CRC advancement by sponging miR-193a-3p and interacting with DDX5, thereby influencing KRAS expression and Wnt/β-catenin signaling.^126^

 Moreover, CRC shows an upregulation of METTL3-dependent m6A methylation, which promotes the processing of miR-181d-5p. This results in increased expression of miR-181d-5p, leading to reduced sensitivity of CRC cells to 5-FU by targeting NCALD.^103^ The m6A methylation of lncRNAs has been identified in 5FU-resistant HCT15 cells, suggesting a role in regulating mRNA expression of drug resistance-associated genes and promoting cancer progression. In particular, the silencing of two specific lncRNAs, ADIRF-AS1 and AL139035.1, associated with MAPK signaling pathways involving FOS, DUSP1, and MEF2C genes has been found to enhance proliferation, metastasis, and potentially regulating drug resistance through.^104^

## Conclusion

 Extensive research has been dedicated to uncovering the molecular pathology of CRC and developing novel epigenetic biomarker assays for accurately diagnosing and predicting the prognosis of this disease. The substantial impact of epigenetic modifications on the onset and advancement of CRC has driven this research focus. Recent findings suggest that abnormal epigenetic alterations and the dysregulation of ncRNAs, including miRNAs and lncRNAs, offer promising avenues for serving as biomarkers in CRC. These biomarkers have the potential to contribute to early detection, prognosis determination, and the identification of therapeutic targets. Further exploration is necessary to fully grasp the role of ncRNA epigenetics in the development of CRC and assess its viability as a diagnostic or prognostic tool for managing CRC effectively.
